# Unsharp masking image enhancement the parallel algorithm based on cross-platform

**DOI:** 10.1038/s41598-022-21745-9

**Published:** 2022-11-23

**Authors:** Yupu Song, Cailin Li, Shiyang Xiao, Han Xiao, Baoyun Guo

**Affiliations:** 1 College of Computer Engineering, Shangqiu Polytechnic, Shangqiu, 476000 Henan China; 2 School of Civil and Architectural Engineering, Shandong University of Technology, Zibo, 255000 Shandong China; 3 Key Laboratory of Geospatial Technology for Middle and Lower Yellow River Regions (Henan University), Ministry of Education, Kaifeng, 475004 Henan China; 4 School of Civil Engineering, Southeast University, Nanjing, 211189 Jiangsu China; 5 School of Information Science and Technology, Zhengzhou Normal University, Zhengzhou, 450044 Henan China

**Keywords:** Computer science, Software

## Abstract

In view of the low computational efficiency and the limitations of the platform of the unsharp masking image enhancement algorithm, an unsharp masking image enhancement parallel algorithm based on Open Computing Language (OpenCL) is proposed. Based on the analysis of the parallel characteristics of the algorithm, the problem of unsharp masking processing is implemented in parallel. Making use of the characteristics of data reuse in the algorithm, the effective allocation and optimization of global memory and constant memory are realized according to the access attributes of the data and the characteristics of the OpenCL storage model, and the use efficiency of off-chip memory is improved. Through the data storage access mode, the fast computing local memory access mode is discovered, and the logical data space transformation is used to convert the storage access mode, so as to improve the bandwidth utilization of the on-chip memory. The experimental results show that, compared with the CPU serial algorithm, the OpenCL accelerated unsharp masking image enhancement parallel algorithm greatly reduces the execution time of the algorithm while maintaining the same image quality, and achieves a maximum speedup of 16.71 times. The high performance and platform transplantation of the algorithm on different hardware platforms are realized. It provides a reference method for real-time processing of a large amount of data of high-resolution images for image enhancement.

## Introduction

The information transmitted by vision accounts for most of the total information received by humans, and the information receiving medium is mainly based on images. In the different aspects of image acquisition, storage, transmission, and display, all of them may make the image have problems such as low contrast, blurred edges, lack of details, and other problems, and even affect the subsequent image analysis and recognition^1,2^. Therefore, in order to improve the visual quality of the acquired image, it is necessary to enhance the original image, highlight the details of the image, filter noise, and improve the image quality^3^.

Image enhancement technology can be divided into point processing, spatial domain processing, and transform domain processing. Transform domain processing is usually applied to image enhancement under non-real-time conditions because of its high algorithm complexity^4,5^. Histogram equalization is a typical method of point processing. This method is to enhance the image in which the values of image pixels are concentrated in a certain area rather than distributed in the entire value space. The effect is obvious and the implementation is simple. However, when the grayscale of the image is stretched, the noise of the image will be magnified at the same time. Image enhancement processing is carried out in the spatial domain, which includes three types of regions, namely, flat regions, detail regions, and edge regions^6–8^. Among them, the detail region and the edge region of the image contain important information of the image. The main purpose of image enhancement processing is to appropriately enhance and sharpen these two types of regions and to avoid magnifying image noise as much as possible. Laplace enhancement is a typical spatial image enhancement algorithm, but this method is very sensitive to image noise. When the contrast in the image is low and the pixels are gathered on a small amount of grayscale, the unsharp masking (UM) processing should be used^9^. The unsharp masking algorithm is an easy to control and effective contour detail enhancement algorithm, which uses the idea of subtraction to indirectly improve the high-frequency information of the image. The basic principle is that the original image subtracts the low-pass filtered image to obtain the high-frequency part, and the high-frequency part is multiplied by a gain coefficient and then added to the original image. This method effectively enriches the edge detail information of the original image. At present, most of the algorithms are implemented in the serial way, and the complex calculation process and the excessive amount of calculation make the real-time effect of image processing poor, which cannot meet the needs of practical applications^10–12^.

How to solve the problem of high performance and low power consumption when processing massive image datasets, which undoubtedly puts forward higher requirements for image processing technology. Although special hardware such as the special chip, Field Programmable Gate Array (FPGA), and Digital Signal Processing (DSP) can be used to optimize algorithm performance, but the costs of research and development are relatively high. Using Graphics Processing Unit (GPU) to accelerate general-purpose computing is a new optimization scheme in heterogeneous computing platforms in recent years, which can reduce additional hardware costs and power consumption. It has great performance advantages in parallel accelerated computing, especially floating-point computing. GPU can provide tens or even hundreds of times higher computing performance than CPU, so it has a high application value. The Khronos Group has launched a heterogeneous computing language, Open Computing Language (OpenCL). OpenCL is a new heterogeneous parallel computing language. Using this programming model, all computing resources in heterogeneous computing systems can be called arbitrarily, which provides a full guarantee for software developers to make efficient use of the performance of all computing resources in computer systems. The OpenCL programming model mainly includes an API for coordinating parallel computing among different processors and a programming language based on ISO C99. This programming model has the characteristics of cross-platform and good compatibility, which greatly facilitates the programming work of developers and promotes the application of GPU parallel computing in various fields.

In this paper, in view of the limitations of the classical unsharp masking algorithm, we consider and propose a method to improve the computing speed of image enhancement by using low-cost and low-power GPU technology. In particular, we consider a method of filtering first and then enhancement. This method has a good effect on the edges and details of the image. This paper has the following two contributions:

1. The UM image processing is realized on GPU by using the OpenCL framework, and it has good data scalability and performance portability. Compared with the performance of serial algorithms, Open Multi-Processing (OpenMP)-based parallel algorithms, and Compute Unified Device Architecture (CUDA)-based parallel algorithms, UM parallel algorithm under the OpenCL architecture achieves an acceleration ratio of 16.71 times, 4.02 times, and 1.11 times, respectively.
2. The standard of performance comparison is diversified. Under the condition that there are few related research results in the field of UM algorithm performance improvement, this paper implements UM image processing on a variety of parallel computing platforms and tests the impact of three parallel modes on the performance of the algorithm. The performance of the UM parallel algorithm accelerated by OpenCL (OCL_UM) is compared with the other two parallel algorithms, and the performance of the algorithm is evaluated objectively as far as possible.

The organization structure of this paper is as follows: “Related research introduction” section mainly introduces the related work, and “Algorithm research and analysis” section is the model analysis of the UM algorithm. It includes the background introduction of OpenCL architecture, the principle of the UM image processing algorithm, and the parallelism analysis of the algorithm. “Problem description” section introduces the migration and optimization of the algorithm on OpenCL and the implementation of GPU parallelism. “Algorithm design and architecture optimization based on OpenCL” section introduces the design and implementation of the algorithm on a variety of parallel computing platforms. “Parallelization based on CPU + GPU heterogeneous computing” section tests the acceleration effect of GPU and the parallel efficiency analysis of a variety of parallel methods. Finally, it summarizes and prospects for the next work.

## Related research introduction

By improving the classical UM algorithm, we can get the high frequency information of different images by modifying the filter, or by modifying the enhancement coefficient, the image enhancement can overcome the problems of noise interference, halo effects, and data overflow, so as to get a good visual effect.

By combining with other methods, the UM algorithm is improved to achieve edge enhancement and noise suppression, so as to improve the image quality. Zhang et al.^13^ improved the UM image enhancement algorithm based on wavelet transform, which has better edge enhancement characteristics. Feng et al.^14^ used the color image as the guide map of the joint bilateral filter to improve the UM depth image enhancement algorithm and improve the quality of the depth image. Fan et al.^15^ proposed an UM image enhancement algorithm based on a singular linear system, which can effectively suppress noise interference and the halo phenomenon. Li et al.^16^ proposed a novel UM image enhancement method guided by Optimum Noticeable Difference (OND), good performance of the OND-UM to enhance the edge and reduce noise sensitivity. Zhang et al.^17^ proposed a novel level-set-based segmentation method with an unsupervised denoising mechanism, which has good robustness and effectiveness. Singh et al.^18^ represented the convolution and pooling layers as the generalized case of filtering and downsampling and implemented an improved depthwise convolution neural network for analyzing the chest X-ray images.

By improving the UM algorithm, the processing efficiency of the algorithm is improved. Wang et al.^19^ proposed an improved UM image enhancement algorithm, and the efficiency of the algorithm is improved. Zhu^20^ proposed an improved self-adaptive UM image enhancement algorithm, which improves the operation speed of the algorithm. Borah et al.^21^ proposed an improved GPU-based UM acceleration algorithm, which effectively suppresses the high-frequency noise background in the image and improves the execution speed of the algorithm.

The UM algorithm is used to improve the performance of the application system on some accelerated platforms. Xu et al.^22^ proposed a novel framework of tool path generation for pocket milling based on image processing, with high effectiveness, easy implementation, and high computing efficiency. Lang et al.^23^ designed a color image detail enhancement algorithm based on quaternion using UM method, and the algorithm has better performance. Sheppard et al.^24^ obtained the segmentation of images of porous and composite materials using a non-sharpening mask, and it has been implemented on cluster-type parallel computers on cluster parallel computers. Yang et al.^25^ presented a method for restoring antialiased edges that are damaged by certain types of nonlinear image filters. It is implemented using GPU technology. Ritschel et al.^26^ applied UM technology to the 3D interactive scene, presented a new approach for enhancing local scene contrast, and realized real-time processing on GPU.

The improved UM algorithm is implemented on the parallel computing platform, and the performance of the algorithm is improved. Xiao et al.^27^ proposed a real-time self-adaptive image enhancement algorithm based on UM and realized real-time image enhancement on FPGA.

At present, most scholars have studied the enhancement of image details and edges, and have proposed an improved UM method. Part of the research is to use the UM algorithm in the application system, using FPGA and GPU to improve the processing speed of the system. There are few research results dedicated to the operational efficiency of UM image enhancement algorithm. However, with the rapid increase of massive image data, how to process the data effectively and quickly has become the focus of the computer research field. This paper takes the UM real-time processing of massive image data as the research object and studies the parallel algorithm of the UM algorithm on GPU platform based on OpenCL heterogeneous parallel computing method. The parallel algorithm is optimized from the aspects of data transmission, vectorized memory access, data parallel computing, and execution configuration. Taking the test image dataset as an example, the accuracy and computational efficiency of the processing results of the serial and parallel methods are compared, and the performance portability of the OCL_UM parallel algorithm is analyzed.

## Algorithm research and analysis

### OpenCL architecture

OpenCL is a standard for writing parallel programs for heterogeneous platforms. The heterogeneous platform is a diverse computing platform, which usually includes CPU, GPU, and other devices with computing power. OpenCL provides a computing framework for task parallelism and data parallelism^28^.

The emergence of OpenCL provides a set of open framework standards for writing parallel programs for heterogeneous platforms composed of multi-core CPU, GPU, and other processors. OpenCL is composed of two parts: the language used to write the running code of the OpenCL device and the API related to the platform. A complete OpenCL program contains logical judgment functions running on CPU and computationally intensive functions (called kernel) running on GPU^29^. Each kernel is executed by multiple work-items at the same time, usually, 32 work-items constitute a warp, which is the basic unit of OpenCL execution. A workspace contains several work-groups, each of which contains several work-items^30^. GPU has its independent memory. Of these, registers only allow the work-items that belong to registers to be accessed, that is, different work-items cannot access registers to each other. Local memory only allows the work-items in the work-group that belongs to local memory to be accessed, that is, work-items located in different work-groups cannot mutually access the local memory. Global memory and constant memory only allow all work-items in the workspace that belongs to global memory to be accessed, but work-items located in different workspaces cannot mutually access global memory and constant memory. The type of memory determines the characteristics such as storage capacity and read and write speed. The global memory has the characteristics of the largest capacity and the highest latency, the local memory, and constant memory have the characteristics of smaller capacity and lower delay, and the register is the memory with the smallest capacity and the fastest access speed^31,32^.

## Problem description

### Principle of linear unsharp masking

The equation of the UM sharpening algorithm is as follows:

1 $$f_{out} \left( {x,y} \right) = f\left( {x,y} \right) + \lambda \times HP\left[ {f\left( {x,y} \right)} \right].$$

The high-pass filter image $HP\left[ {f\left( {x,y} \right)} \right]$ is realized by the transformation processing of the low-pass filter image $LP\left[ {f\left( {x,y} \right)} \right]$, then there is $HP\left[ {f\left( {x,y} \right)} \right] = f\left( {x,y} \right) - LP\left[ {f\left( {x,y} \right)} \right]$, the expression of $HP\left[ {f\left( {x,y} \right)} \right]$ is substituted into (1), and the complete image UM equation is obtained as follows:

2 $$f_{out} \left( {x,y} \right) = f\left( {x,y} \right) + \lambda \times \left\{ {f\left( {x,y} \right) - LP\left[ {f\left( {x,y} \right)} \right]} \right\}.$$

Among them, $f_{out} \left( {x,y} \right)$ is the sharpened image signal, $f\left( {x,y} \right)$ is the original image signal, and $\lambda$ is the enhancement coefficient factor with rotation invariance, which is used to control the degree of detail enhancement. The larger $\lambda$ is, the more obvious the image detail is, and the smaller $\lambda$ is, the closer the output image is to the original image^33–35^. In general $\lambda \,{ \ge }\,0$, $\lambda \,{ = }\,1$ is unsharp masking, and $\lambda \, > \,1$ is a high lifting mask filter. Generally, a reasonable $\lambda$ should be between 0.5 and 1.5 to prevent from overwhelmed sharpening effect. Because $\lambda$ is a constant independent of image content, $f_{out} \left( {x,y} \right)$ is a linear unsharp mask image signal. HP and LP represent sharpening and smoothing filtering processes, respectively. The commonly used spatial sharpening filtering includes Sobel filtering and Laplace filtering, etc. The commonly used spatial smoothing filtering includes median filtering, Gaussian filtering, etc. Different filtering methods can be chosen according to the needs of practical applications. This paper uses Gaussian smoothing filter to realize low-pass filter images $LP\left[ {f\left( {x,y} \right)} \right]$^36–38^.

When $f\left( {x,y} \right)\,\, - \,\,LP\left[ {f\left( {x,y} \right)} \right]\, > \,0$, that is $HP\left[ {f\left( {x,y} \right)} \right]\, > \,0$, so there is $f_{out} \left( {x,y} \right)\, > f\left( {x,y} \right)$. When $f\left( {x,y} \right)\, - \,LP\left[ {f\left( {x,y} \right)} \right]\, < \,0$, that is $HP\left[ {f\left( {x,y} \right)} \right]\, < \,0$, so there is $f_{out} \left( {x,y} \right)\, < \,f\left( {x,y} \right)$. Through the analysis, it is known that this method can make the brighter area of the original image brighter and the darker area darker, making the edge region more obvious, so it achieves the purpose of image sharpening^39^.

### Gaussian filter

Gaussian filtering is a weighted mean filtering method. The Gaussian filter is a low-pass filter that selects the weights according to the shape of the Gaussian function and is very effective in suppressing the noise which obeys the normal distribution. The principle of the Gaussian filter is to use a Gaussian convolution template to scan each pixel of the image one by one and perform convolution operations on the template and the pixels it covers, so as to achieve the purpose of smoothing noise.

The characteristic of Gaussian distribution is that the probability on both sides of the mean is very large, and the farther away from it, the smaller the probability. Therefore, the idea of the Gaussian function in filtering is the closer the point to a certain pixel point, the greater the impact on it, let its weight be greater, and the farther the point, the smaller the impact on it, let its weight smaller^40^. Image denoising needs to calculate the neighborhood of $\left( {2k + 1} \right)\, \times \,\left( {2k + 1} \right)$ size near the center point of each pixel in the image. In the field of image processing, the discrete Gaussian function with a two-dimensional zero mean is used for smoothing filtering. The function expression is as follows.

3 $$G\left( {x,y} \right)\, = \,\frac{1}{{2\pi \sigma^{2} }}e^{{ - \frac{{x^{2} \, + \,y^{2} }}{{2\sigma^{2} }}}} \,\,\left( { - k\, \le \,x,\,y\, \le \,k} \right).\,$$

Among them, $\left( {x,y} \right)$ represents pixel coordinates, $\sigma$ the standard deviation is a Gaussian distribution parameter, and the width of the Gaussian function is determined by $\sigma$.

The Gaussian template discretizes the two-dimensional continuous normal distribution function to obtain a $\left( {2k + 1} \right)\, \times \,\left( {2k + 1} \right)$-order weight matrix, as shown in Eq. (4)^41,42^.

4 $$G\left( {i,j} \right)\, = \,\frac{1}{{2\pi \sigma^{2} }}e^{{ - \frac{{i^{2} \, + \,j^{2} }}{{2\sigma^{2} }}}} \,\,\,\,\left( { - k\, \le \,i,\,j\, \le \,k} \right)\,\,.$$

**Figure 1:**
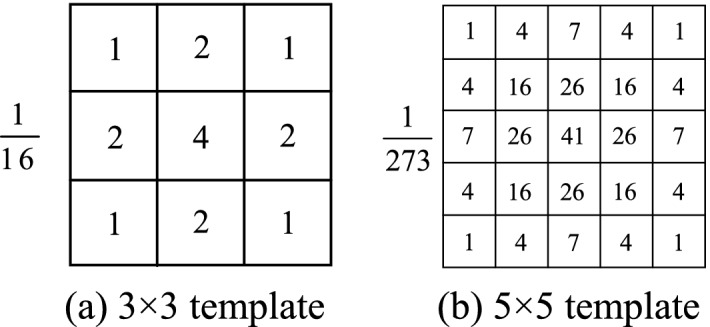
Gaussian convolution kernel.

After constructing the convolution kernel, the convolution kernel needs to be normalized, that is, the values in the whole Gaussian convolution kernel are accumulated, and each value in the convolution kernel is divided by the cumulative value. The integer Gaussian kernel with a standard deviation of 1.0 is shown in Fig. 1^43^.

To sum up, from Eqs. (2) and (4), the calculation equation of the UM algorithm is as shown in Eq. (5):

5 $$f_{out} \left( {x,y} \right)\, = \,f\left( {x,y} \right)\, + \,\lambda \, \times \,\left\{ {f\left( {x,y} \right)\, - \,G\left( {i,j} \right)\, \times f\left( {x,y} \right)} \right\}.$$

### Image boundary processing

Due to the existence of the Gaussian filter template width, if there are no extended pixels at the edge of the image, it will not be possible to perform UM processing on the pixels at the edge of the image, so the UM processing for the whole image is incomplete. At the same time, it is necessary to perform complex logical judgment operations on edge pixels, which is not conducive to parallel image processing. In addition, if the edge pixels are not displayed in the image processing result in order to facilitate parallel computing processing, the size of the resulting image will be reduced. And with the increase of the width of the Gaussian filter template, the size of the resulting image will become smaller and smaller. These handling methods are unreasonable. Therefore, for the image size is $H{ \times }H$ and the convolution template is $n\,{ \times }\,n$, it is necessary to expand and fill the pixel values around the edge points of the image. The width and height of the expanded image are both $H\, + \,n\, - \,1$. In this way, when the OpenCL kernel follows the same logic and computing path to process the entire image, the additional checks needed to process the image boundaries are avoided, and GPU is more efficient.

### Algorithm hotspot analysis

Before designing the UM parallel algorithm based on GPU, we should first profile the algorithm to find the hotspots of the algorithm. CPU is Intel Core six-core i5 9400F, the image size of the test image is 7682 × 8182, and the Gaussian filter template size is $3\, \times \,3$. Use float as the data element type. In order to ensure that the test time is more accurate, the average value of 20 running results is taken as the test result of the experiment when recording the results. The time of each calculation process of the UM algorithm and its proportion in the total calculation time are shown in Table 1.

**Table 1:** Running time and proportion of each step of the image UM algorithm.

Main calculation steps	Execution time/ms	Time percentage/%
Read in image data	768.30	13.45
Extended image	974.42	17.06
Gaussian template calculation	0.53	0.03
Unsharp masking processing	2705.50	47.39
Output image enhancement results	1260.55	22.07
Total	5709.30	100.00

It can be seen from the data in Table 1 that in the calculation process, the proportion of UM processing time in the calculation process is 47.39%, which is the largest step in the amount of calculation. Gaussian blur needs to take a $n\,{ \times }\,n$ window around the calculation point and take the calculation point as the center to calculate the convolution in this window, as well as the extraction, enhancement, and superposition of the high-frequency components of the corresponding image. Then, the UM processing in turn at each point in the two-dimensional data will result in a large amount of computation, so reducing the computing time overhead of the UM processing is the main problem that the algorithm needs to solve.

In the unsharp masking algorithm, it is assumed that the image size is $H \times H$ and the Gaussian convolution template size is $n\,{ \times }\,n$. Then the time complexity of the extended image step is ${\rm O}\left( {H^{{2}} } \right) + {\rm O}\left( {H \times n} \right)$, the time complexity of calculating the Gaussian template step is ${\rm O}\left( {n^{{2}} } \right)$, and the time complexity of the unsharp masking processing step is ${\rm O}\left( {H^{{2}} n^{{2}} } \right)$. The total time complexity of the unsharp masking algorithm is ${\rm O}\left( {H^{{2}} n^{{2}} } \right) + {\rm O}\left( {H^{{2}} } \right) + {\rm O}\left( {H{ \times }n} \right) + {\rm O}\left( {n^{{2}} } \right)$. Since *H* is much larger than *n*, the total time complexity of the unsharp masking algorithm is ${\rm O}\left( {H^{{2}} n^{{2}} } \right)$. It can be seen that the main computational load of the algorithm lies in the unsharp masking processing.

### Parallel feature analysis of algorithms

**Figure 2:**
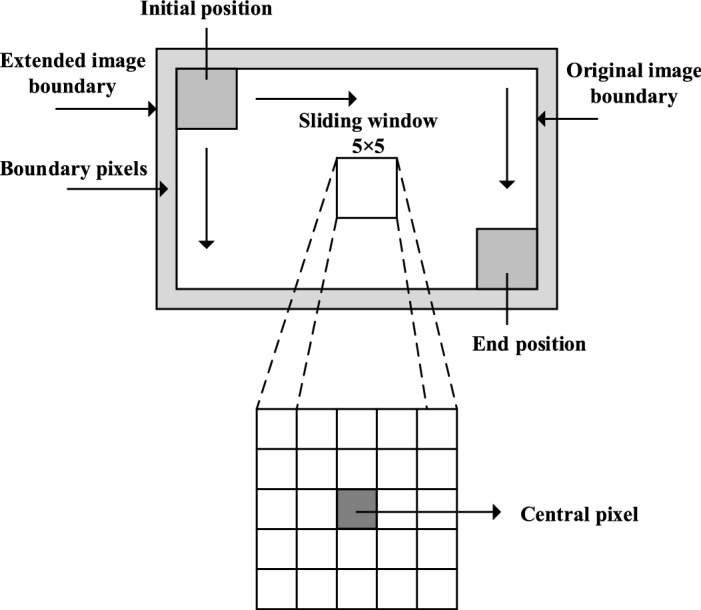
Image with the sliding window.

The processing process of low-frequency components obtained by spatial low-pass filtering in the UM image enhancement algorithm is shown in Fig. 2. The Gaussian filter window slides down one bit until its central pixel reaches the last line of the image. Continue this process by moving the sliding window to the right to the top of the next column to process the pixels of the next column, and repeat the process until the last pixel in the image is reached. Through the analysis of the calculation process of two-dimensional convolution, it is found that there are a large number of multiplicative and cumulative calculations in the whole calculation process and the data are discrete and independent.

According to the UM image enhancement algorithm represented by Eq. (5), each pixel of the processed image needs to be traversed. The same operation is performed for each pixel, that is, the Gaussian filtering of the corresponding pixel and the extraction, enhancement, and signal superposition of the high frequency components of the image are calculated. According to the above analysis, it can be found that the UM algorithm has high computational complexity, and the serial implementation method is less real-time for high-resolution images. In this algorithm, the operation between pixels is relatively independent, the degree of data dependence is low, and it has high inherent parallelism, so it is suitable for GPU parallel implementation.

Therefore, OpenCL can be used to accelerate these operations in parallel. A large number of work-items are created in the OpenCL acceleration system, and each work-item performs the unsharp masking processing on the corresponding pixel. Because all work-items perform the same calculation process at the same time, the time complexity of the unsharp masking algorithm will be reduced to ${\rm O}\left( {n^{{2}} } \right)$, which is a very low level. If all pixels are not processed in one kernel function, each work-item will execute the unsharp mask kernel function at least ${{\left( {H^{{2}} n^{{2}} } \right)} \mathord{\left/ {\vphantom {{\left( {H^{{2}} n^{{2}} } \right)} {tsum}}} \right. \kern-\nulldelimiterspace} {tsum}}$ times, where $tsum$ is the number of work-items. In this case, the time complexity will be ${\rm O}\left( {{{\left( {H^{{2}} n^{{2}} } \right)} \mathord{\left/ {\vphantom {{\left( {H^{{2}} n^{{2}} } \right)} {tsum}}} \right. \kern-\nulldelimiterspace} {tsum}}} \right)$. It is important to note that because there are a large number of active work-items that can be maintained in GPU, that is, $tsum$ is always a large value. Therefore, the time complexity ${\rm O}\left( {{{\left( {H^{{2}} n^{{2}} } \right)} \mathord{\left/ {\vphantom {{\left( {H^{{2}} n^{{2}} } \right)} {tsum}}} \right. \kern-\nulldelimiterspace} {tsum}}} \right)$ of the unsharp masking parallel algorithm is much smaller than the time complexity ${\rm O}\left( {H^{{2}} n^{{2}} } \right)$ of the serial algorithm.

## Algorithm design and architecture optimization based on OpenCL

### Parallel algorithm description

Because the calculation of UM image enhancement is complex and the image data to be processed are independent of each other, the pixel points are also processed separately one by one. Therefore, UM calculations can be performed on data based on the powerful parallel computing capabilities of the GPU. The work-item is logical one-to-one corresponding to the calculation of each pixel, and the UM calculation part is designed as a kernel function executed on GPU. As a result, the speed of data processing is accelerated and the processing time of UM is reduced. But in fact, the image data has a relatively large amount of data, so the number of work-items is consistent with the length of one-dimensional data in the $x$-axis direction while ensuring the coalesced memory access of the global memory. The overall parallelization idea of UM image enhancement is described as follows.
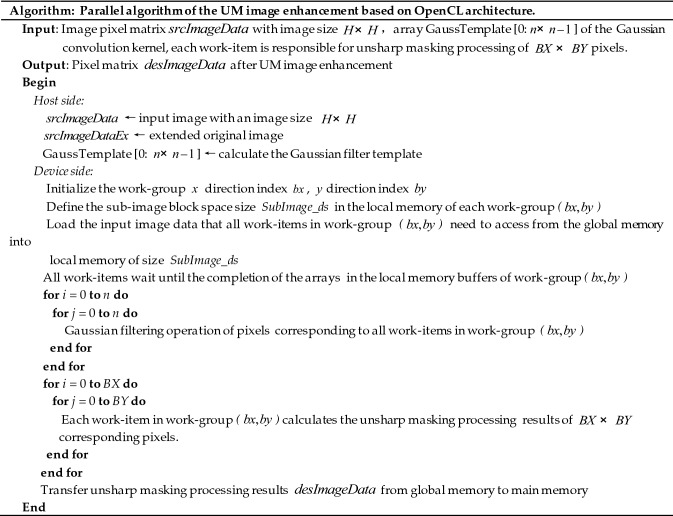


### Method design

**Figure 3:**
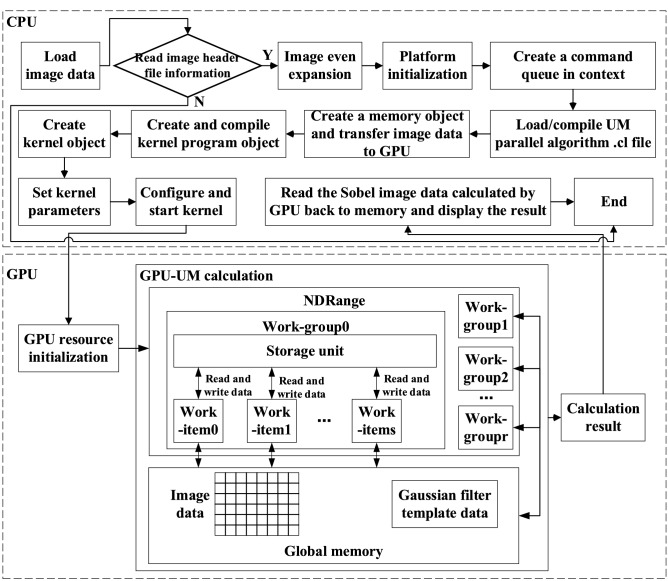
Parallel computing step diagram of the UM image enhancement based on OpenCL.

The implementation of the UM image enhancement parallel algorithm mainly consists of two parts: the host side and the device side. The main flow of the parallel algorithm is shown in Fig. 3.

The main implementation steps of UM image enhancement parallel computing are as follows.

From the above analysis, for the calculation of memory consumption, the operation time will be much lower than the memory copy time. Therefore, due to the special structure of the hardware and the support of the operating environment, the use of shared memory will avoid a large number of duplicated data copies. The specific implementation steps are as follows:

- *Step1* Acquire the number of computing devices available on the current platform and the corresponding device parameters.
- *Step2* Create a context under the platform, including the devices under the current platform.
- *Step3* Initialize the corresponding GPU device and create a command queue.
- *Step4* The CPU side calls the clCreateBuffer() function to allocate global memory and allocates buffers in global memory.
- *Step5* Call the clEnqueueMapBuffer() function to map the buffer.
- *Step6* Fill the contents of the global memory buffer with image data.
- *Step7* Host puts the kernel function into the command queue, and then calls the clSetKernelArg() function to pass the global memory buffer to the kernel function.
- *Step8* Execute the kernel function according to the assigned task.
- *Step9* In OpenCL parallel computing, work-items fetch data from the global memory to the local memory according to the corresponding calculated pixels, then synchronize the work-items, and wait for all work-item operations to be completed. Inside the kernel function, the image data is stored in the local memory in the form of a two-dimensional matrix, and multiple work-items are organized for operation in the form of a two-dimensional work-group. In the UM algorithm, each pixel only needs to participate in the convolution calculation together with eight adjacent pixels. In the kernel function, the constant memory is used for the Gaussian filter template data stored in GPU to speed up the data access speed.
- *Step10* The image data processed by UM are transferred to CPU memory and written to a file to form the processed image.

## Parallelization based on CPU + GPU heterogeneous computing

### Parallel scheme design

The algorithm design in the image parallel processing mode needs to consider the mapping between the image data matrix and the processor set. The general criteria for implementing mapping are to improve the parallelism of algorithm execution (the purpose is to improve the utilization of compute units) and to explore data correlation (the purpose is to avoid the communication overhead), so as to improve the execution efficiency of the algorithm. Based on the parallel design of the algorithm under the OpenCL architecture, the key works are to allocate data to work-items and to adopt a simple way to implement work-items corresponding to the data.

Based on the above analysis, the principles to be followed in the design of the OCL_UM image enhancement parallel algorithm: ① For the parallelism of the mining algorithm, fine-grained parallelism is adopted to improve the utilization rate of compute units. ② According to the low coupling between image data processing, the correlation between work-item processing image data is reduced, and the communication overhead between work-items is reduced. ③ Reasonably organize the workspace and divide the image data processing structure reasonably, so that the work-item can locate the data processed through the work-item index.

**Figure 4:**
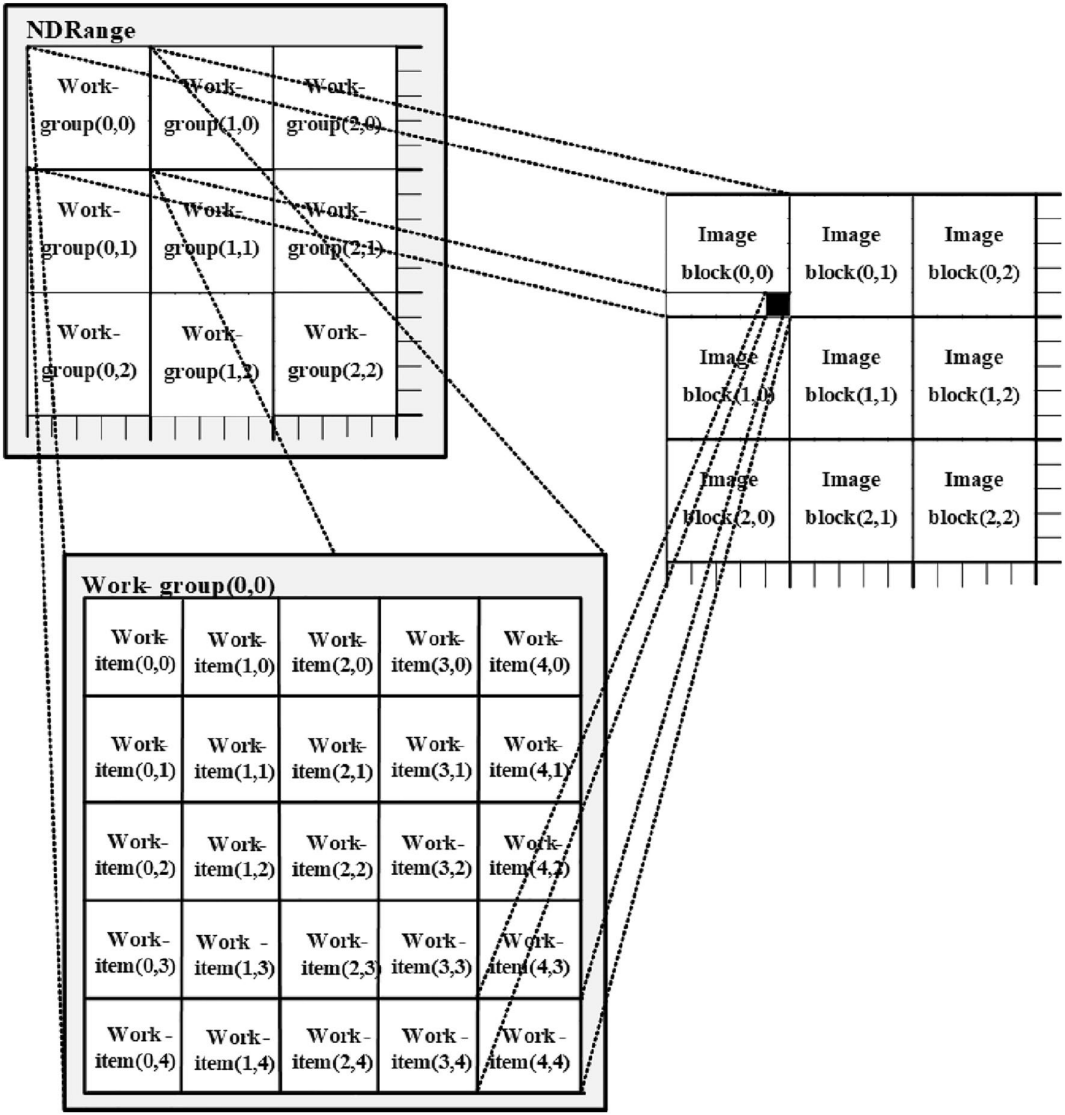
Correspondence relationship diagram between work-items and pixels.

In the process of calculation, most of the UM algorithms take the image pixels as individuals. Single Instruction Multiple Thread (SIMT) data parallel computing mode is adopted in the design of a parallel algorithm according to the characteristics of computing instructions. Logically, a pixel can be made to correspond to a work-item in GPU, and one work-item is responsible for the UM processing of a pixel, in order to achieve the purpose of parallel computing, as shown in Fig. 4. At the same time, although the processing between different pixels does not need to communicate during the calculation process, the number of work-items in each work-group has no effect on the calculation results. However, it has a significant impact on the execution efficiency of GPU, so the best kernel configuration method should be chosen.

### Pixel coordinate transformation

According to the characteristic that convolution multiplication-addition is independent of each other in the convolution operation of the Gaussian low-pass filter, the most general idea is to calculate a convolution multiplication-addition by one work-item to realize convolution parallelism. In the UM image enhancement algorithm, each work-item will be responsible for the convolution multiplication-addition of the neighborhood of a pixel and the Gaussian convolution kernel template. The specific description of the data structure involved in the algorithm after mapping to OpenCL is shown in Eq. (6), which is addressed through the two-dimensional index ID of the work-group in NDRange and the two-dimensional index ID of the work-item in the work-group.

6 $$\begin{gathered} tidx\,{ = }\,get\_local\_id{(0)} \,{ + } \,get\_group\_id{(0)} \times get\_local\_size{(0),} \hfill \\ tidy\,{ = }\,get\_local\_id{(1)} \,{ + } \,get\_group\_id{(1)} \times get\_local\_size{(1),} \hfill \\ \end{gathered}$$

where $get\_local\_id{(0)}$ and $get\_local\_id{(1)}$ are the index numbers of the work-item in the $x$ and $y$ directions in the work-group, $get\_group\_id{(0)}$ and $get\_group\_id{(1)}$ are the index numbers of the work-group in the $x$ and $y$ directions in the workspace, and $get\_local\_size{(0)}$ and $get\_local\_size{(1)}$ are the dimensions of the work-group in the $x$ and $y$ directions, respectively. $tidx$ and $tidy$ represent the one-dimensional index marks of a work-item in the work-group in the $x$ and $y$ directions in the workspace. Therefore, each work-item can use ${(}tidx,tidy{)}$ coordinates to find the corresponding pixel position, so as to determine the coordinate region of the pixel neighborhood for convolution operation with the Gaussian convolution kernel.

## Performance tuning

### Multi-point access optimization

**Figure 5:**
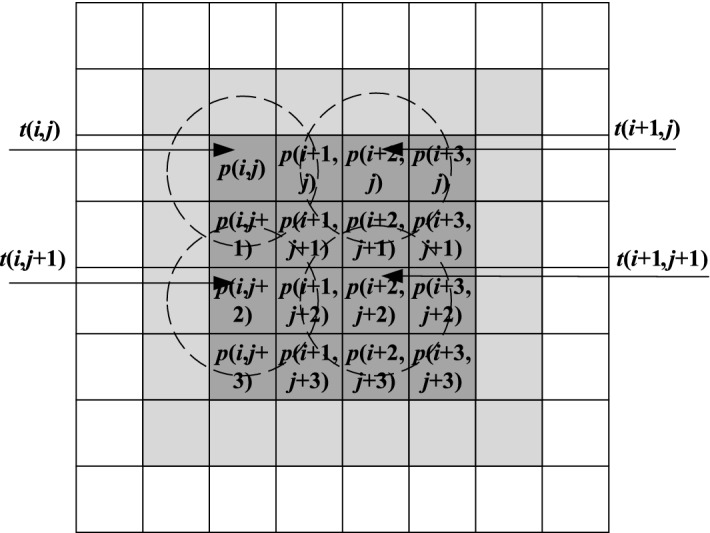
Multi-point access within a work-group.

If the size of the Gaussian convolution kernel is ${3} \times {3}$, it can see from Fig. 5 that for each output point processed by UM image enhancement, nine data need to be read from the global memory. For example, take the output pixel $p{(}i{,}j{)}$ as the center of the data in the dashed circle. These data are discarded after the calculation of the current point, and the calculation of the latter point needs to retrieve the points from the global memory again. Among the 36 data needed to process 16 pixels, it generally needs to be read repeatedly for 1–9 times, so the access efficiency is low. At the same time, because it takes about 400–600 clock cycles for the system to access image data from the global memory, and only 1–16 clock cycles for accessing image data directly from the local memory, the access efficiency of the GPU will be greatly improved. Therefore, according to the cascading characteristics of convolution operation access to memory, a multi-point access technique is designed to improve the access efficiency of image data: the work-item $t{(}i{,}j{)}$ in Fig. 5 processes four pixel output points of $p{(}i{,}j{)},\,p{(}i{ + 1,}j{)},\,p{(}i{,}j{ + 1)}$ and $p{(}i{ + 1,}j{ + 1)}$, and so on. Accordingly, the system reads the 36 image data related to the output pixels that need to be processed by all work-items in the work-group (including 4 work-items) at one time and places them in the local memory, and the post-point calculation can repeatedly access the front points stored in the local memory. In this way, not only the repeated access to global memory is avoided, but also the vectorization processing of the output points can be realized, and the memory access efficiency is improved. The multi-point technology access method is shown in Fig. 5.

### Memory optimization

The memory in GPU can be divided into different types and have different characteristics. The correct and reasonable use of memory will greatly improve the performance of the system. The image data in the current algorithm are read from the global memory. In order to further improve the performance of the algorithm, the constant memory optimization algorithm can be used to transfer the system performance bottleneck from the global memory to the L2 cache. Constant memory has the characteristics of fast memory access and low latency, and the information stored in it cannot be modified. And it has the access characteristic of global memory, which can be accessed by all work-items in the workspace.

The Gaussian convolution kernel in the UM image enhancement algorithm is constant. These constants will not change in the whole calculation process of the algorithm and have a read-only characteristic. Therefore, the Gaussian convolution kernel is suitable to be placed in constant memory. In this way, the transfer of template data from global memory to constant memory can greatly increase the speed of reading and writing. It should be noted that the GPU has a limit on the capacity of the constant memory, so the amount of data put into the constant memory cannot exceed its maximum limit. However, the storage capacity of convolution kernel data is generally small and will not exceed the capacity limit of constant memory.

### Work-group dimension optimization

Different work-group dimensions will result in different concurrency of work-items. The system can achieve the best performance by setting the work-group dimension reasonably. Therefore, the work-group dimension is an important factor affecting the performance of the algorithm. The warp size of the GPU is 32, so the work-group’s dimension should be an integral multiple of 32. At the same time, the GPU constraint value for the number of work-items contained in the work-group is 1024. Therefore, this paper compares the performance of OCL_UM image enhancement operation time when the image size is 7682 × 8182 and the work-group dimensions are 8 × 8, 16 × 16, 24 × 24, 32 × 32, respectively. It can be found from Table 2 that when the work-group dimension is set to 16 × 16, the calculation time is the shortest.

**Table 2:** The influence of work-group dimension on performance.

Number of work-items in the work-group	Computing time (ms)
8 × 8	170.89
16 × 16	167.94
24 × 24	178.09
32 × 32	174.58

## Other parallel solutions

### UM algorithm based on OpenMP

When using OpenMP to carry out the UM algorithm (OMP_UM) for multi-core parallel computing, the thread quantity should be created based on the number of CPU cores $r$. Each thread is assigned to the target block of the algorithm in a self-heuristic way, and the ${{H^{{2}} } \mathord{\left/ {\vphantom {{H^{{2}} } r}} \right. \kern-\nulldelimiterspace} r}$-time UM image sharpening of the target block is calculated serially. Each OpenMP thread is responsible for the convolution operation between the neighborhood of a pixel and the Gaussian convolution kernel, as well as the UM sharpening processing of a pixel.

The parallel model of OpenMP is based on the Fork-Join form, and the execution area between Fork and Join is regarded as a parallel region. The main thread of the system will encounter the parallel structure instructions at the beginning of the UM image sharpening processing section, open the parallel area, and then create a thread group. Each thread in the thread group executes in parallel in the next parallel execution area, that is, the Fork action. When all threads in the system finish the UM image sharpening work in parallel, exit the parallel structure. Then they are concatenated together, only the main thread continues to execute, and the other threads end, ending the current parallel region, that is, the Join action.

### UM algorithm based on CUDA

The most basic unit of work in CUDA is the thread. In the CUDA-based UM algorithm (CUDA_UM), a thread is assigned to UM image sharpening processing for each pixel. Each thread performs the convolution operation of calculating the neighborhood of a pixel and the Gaussian filter template and can complete the UM sharpening processing of a pixel. In this way, when the image size is $H \times H$, the number of threads allocated in theory is $H \times H$. Then the size $blockDim{.}x \times gridDim{.}x$ of the computing grid is set to $H \times H$, and each thread index in the global index corresponds to the coordinates of 1 pixel in the image. Find the global ID of the corresponding thread in the two-dimensional space of the image, and use $blockDim{.}x \times blockIdx{.}x + threadIdx{.}x$ and $blockDim{.}y \times blockIdx{.}y + threadIdx{.}y$ split double loops to form a kernel inner loop.

Thread blocks are organized at a higher level than threads, and threads of the same thread block can execute concurrently on a streaming multiprocessor. In this paper, the dimension size of the grid needs to be determined according to the current original image size. The dimension size of the thread block is usually a multiple of 16, and the maximum is 1024. It is represented as 16 × 16 in a two-dimensional organization. The CUDA_UM parallel algorithm adopts an optimization method similar to that of the OCL_UM parallel algorithm, which will not be repeated in view of the space.

## Performance evaluation and analysis

### Test environment

#### The platform 1

The CPU is Intel Core six-core i5 9400F, and the main frequency is 2.9 GHz. The system memory is DDR4 with a transmission bandwidth of 19.2 GB/s. The memory capacity is 8 GB, the operating frequency is 2666 MHz, the memory bandwidth is 12.27 GB/s, and the graphics card adopts NVIDIA Geforce GTX1060, which belongs to Pascal architecture. GTX1060 has 1152 CUDA cores, computing power 6.1, core frequency 1506 MHz, with 3 GB GDDR5 memory capacity, memory bit width 192 bits, and video memory bandwidth 192 GB/s.

#### The platform 2

CPU is AMD Ryzen 5 3600 with six cores and the main frequency is 3.6 GHz. The system memory is DDR4, with 8 GB memory capacity and 3200 MHz working frequency, and the graphics card uses Radeon RX 5700 XT, with 2560 stream processors, core frequency 1605 MHz, with 4 GB GDDR6 video memory capacity, memory bit width 256 bits, video memory bandwidth 448 GB/s.

The operating system is Microsoft Windows 10 64-bit, the GPU application development software is CUDA 11.0, and the development environment is Microsoft Visual Studio 2015.

## Image quality evaluation

### Subjective evaluation

**Figure 6:**
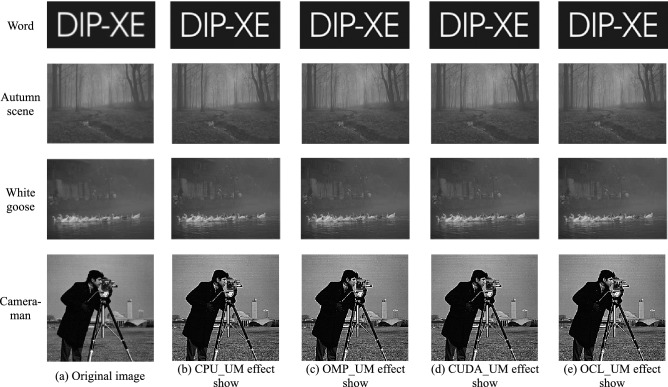
Unsharp masking image enhancement effect shows.

Figure 6a is the original image, and Fig. 6b–e are the images processed by CPU_UM, OMP_UM, CUDA_UM, and OCL_UM systems, respectively. The enhancement effects of multiple algorithms can be compared intuitively from Fig. 6. After UM image enhancement, the effect is obvious. Compared with the original image, the detail area of the image is effectively enhanced and the contrast is improved. In particular, the silhouette of the cameraman and the texture of the camera, text edges, and other details are better reflected. The outline of the white goose body and the background house is clear, and the branches are clearly visible in the autumn scene. Moreover, there is no obvious over enhancement problem, and the enhancement effect is basically the same.

From the processing results of Fig. 6, the serial processing results of Fig. 6b, and the parallel processing results of Fig. 6c–e have obtained relatively clear image enhancement images, and no difference can be distinguished by the naked eye.

### Objective comment

Because the original image contains more details, the entropy value of the image before and after processing is detected. One-dimensional entropy can explain the amount of information contained in the aggregation of grayscale distribution in the image: the more information the image contains, the greater the entropy value. Information entropy is defined as:

7 $$E = - \sum\limits_{{i{ = 0}}}^{{L - {1}}} {P_{i} {log}_{{2}} P_{{i}} } .$$

In Eq. (7),$P_{i}$ is the gray histogram value of the image, that is, $P_{i} = {{N_{i} } \mathord{\left/ {\vphantom {{N_{i} } N}} \right. \kern-\nulldelimiterspace} N}$, $N_{i}$ represents the number of pixels whose gray value is $i$, $N$ is the total number of pixels in the image, and $L$ is the total gray level number of the image. In order to compare the enhancement effects of the UM image more comprehensively and accurately, the image information entropy lists before and after serial/parallel algorithm processing are compared. Enter the same test image, set the same parameters, and compare them with five cases, as shown in Table 3.

**Table 3:** Comparison of image information entropy in different UM algorithms.

Approach	Word	Autumn scene	White goose	Cameraman
No processing	2.74	6.50	6.06	6.85
CPU_UM	3.02	6.87	6.39	7.02
OMP_UM	3.02	6.87	6.39	7.02
CUDA_UM	3.02	6.87	6.39	7.02
OCL_UM	3.02	6.87	6.39	7.02

It can be seen from Table 3 that the image information entropy value of the four UM images enhanced by CPU_UM, OMP_UM, CUDA_UM, and OCL_UM is higher than that of the original image, the amount of image information is increased, and the overall image contrast is significantly improved. It shows that the enhancement effect of UM is relatively obvious, which is consistent with the actual subjective evaluation conclusion. At the same time, the image information entropy of the four UM enhancement algorithms is the same, which explains the correctness and effectiveness of the OCL_UM image enhancement parallel algorithm proposed in this paper.

## Experimental data analysis

### Algorithm operation time analysis

Because there are both non-parallelization and parallelization parts in the parallel algorithm design, the statistical analysis of the overall computing speed of the algorithm cannot accurately reflect the ability of the GPU to accelerate the execution of the algorithm. Therefore, taking the function of the UM processing part as the object, the running time of the algorithm part is counted. At the same time, the serial algorithm CPU_UM with the same function and the same parameters is implemented and the running time is counted, so as to analyze the acceleration performance of the OCL_UM parallel algorithm.

First of all, a batch of images of different sizes are generated through the image editing software tool Photoshop. The specific image resolutions are 525 × 525, 750 × 750, 978 × 1024, 1893 × 2048, 3877 × 4096, 7682 × 8182, and 16,364 × 8182, a total of 7 groups experimental data. Then the 3 × 3 Gaussian convolution kernel is used to compare and test the speed of the four serial/parallel algorithms CPU_UM (the platform 1), OMP_UM (the platform 1), CUDA_UM, and OCL_UM to process these images. Because these different algorithms achieve the same way of reading/writing image files and run on the CPU side. Therefore, the time consumed by the read/write of the image file and the non-parallelizable part of the algorithm can be ignored. The comparison of the time consumed by the four UM serial/parallel algorithms is shown in Table 4, in which the time data is obtained by averaging the algorithm after multiple tests.

**Table 4:** Time-consuming comparison of UM algorithm.

Image size (px)	CPU_UM (ms)	Parallel processing time (ms)			
		OMP_UM	CUDA_UM	OCL_UM (AMD)	OCL_UM (NVIDIA)
525 × 525	11.91	3.43	1.53	1.42	1.41
750 × 750	24.58	7.00	1.94	1.75	1.74
978 × 1024	45.10	11.99	3.23	3.14	3.07
1893 × 2048	178.50	46.85	12.73	12.03	11.95
3877 × 4096	631.60	160.71	41.72	41.69	41.66
7682 × 8182	2705.50	626.70	170.26	168.88	167.94
16,364 × 8182	5312.00	1171.00	318.00	317.92	317.85

**Figure 7:**
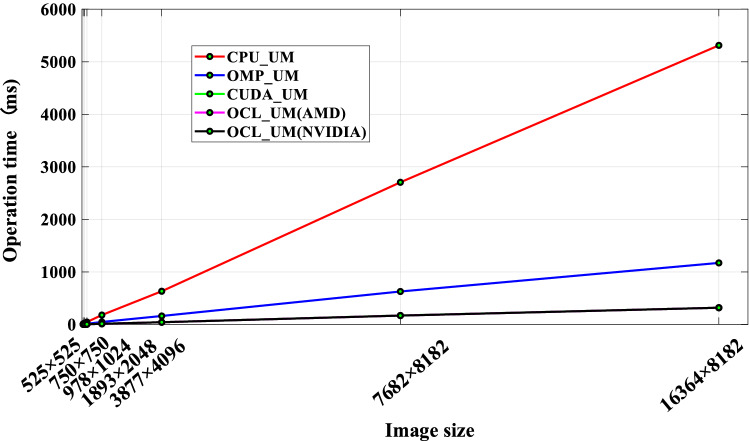
Running time comparison of the image dataset.

According to Table 4, draw a line chart, as shown in Fig. 7. In order to show the execution time of the UM image enhancement algorithm on the serial platform and different parallel platforms more intuitively. From the figure, it is shown that the computing time of OMP_UM, CUDA_UM, and OCL_UM parallel algorithms is significantly lower than that of CPU_UM serial algorithms. Especially in CUDA_UM and OCL_UM parallel algorithms, with the increase of image size, the number of work-items started in the algorithm increases, and the utilization of computing resources increases. The running time of the UM parallel algorithm based on the GPU is greatly reduced, and the execution speed of the algorithm is improved obviously. In the relevant research section of this manuscript, it has been pointed out that although some achievements are applied using the UM algorithm, there are no results that can directly compare the performance of the UM algorithm.

## Algorithm speedup analysis

### GPU accelerated performance analysis

In parallel algorithms, the speedup is a more direct performance evaluation index, which reflects the ability of parallel algorithms to improve performance when solving practical problems.

#### Definition 1

The ratio of the execution time $T_{{CPU{\_}UM}}$ of the CPU_UM serial algorithm to the execution time $T_{{OMP{\_}UM}}$ of the OMP_UM parallel algorithm is called the acceleration ratio $S_{OMP}$, as shown in Eq. (8).

8 $$S_{OMP} = \frac{{T_{{CPU{\_}UM}} }}{{T_{{OMP{\_}UM}} }}.$$

#### Definition 2

The ratio of the execution time $T_{{CPU{\_}UM}}$ of the CPU_UM serial algorithm to the execution time $T_{{CUDA{\_}UM}}$ of the CUDA _UM parallel algorithm is called the acceleration ratio $S_{CUDA}$, as shown in Eq. (9).

9 $$S_{CUDA} = \frac{{T_{{CPU{\_}UM}} }}{{T_{{CUDA{\_}UM}} }}.$$

#### Definition 3

The ratio of the execution time $T_{{CPU{\_}UM}}$ of the CPU_UM serial algorithm to the execution time $T_{{OCL{\_}UM}}$ of the OCL_UM parallel algorithm on the corresponding GPU platform is called the acceleration ratio $S_{OCL}$, as shown in the Eq. (10).

10 $$S_{OCL} = \frac{{T_{{CPU{\_}UM}} }}{{T_{{OCL{\_}UM}} }}.$$

#### Definition 4

The ratio of the execution time $T_{{OMP{\_}UM}}$ of the OMP_UM parallel algorithm to the execution time $T_{{OCL{\_}UM}}$ of the NVIDIA GPU-based OCL_UM parallel algorithm is called the relative acceleration ratio $RS_{OMP - OCL}$, as shown in Eq. (11).

11 $$RS_{OMP - OCL} = \frac{{T_{{OMP{\_}UM}} }}{{T_{{OCL{\_}UM}} }}.$$

#### Definition 5

The ratio of the execution time $T_{{CUDA{\_}UM}}$ of the CUDA_UM parallel algorithm to the execution time $T_{{OCL{\_}UM}}$ of the NVIDIA GPU-based OCL_UM parallel algorithm is called the relative acceleration ratio $RS_{CUDA - OCL}$, as shown in Eq. (12).

12 $$RS_{CUDA - OCL} = \frac{{T_{{CUDA{\_}UM}} }}{{T_{{OCL{\_}UM}} }}.$$

From Table 4, the speedups of OMP_UM, CUDA_UM, and OCL_UM parallel algorithms on each group of test images are shown in Table 5.

**Table 5:** Accelerated results comparison.

Image size (px)	Acceleration ratio				Relative acceleration ratio	
	*S*_*OMP*_	*S*_*CUDA*_	*S*_*OCL*_ (AMD)	*S*_*OCL*_ (NVIDIA)	*RS*_*OMP*-*OCL*_	*RS*_*CUDA*-*OCL*_
525 × 525	3.47	7.78	8.39	8.45	2.43	1.09
750 × 750	3.51	12.67	14.05	14.13	4.02	1.11
978 × 1024	3.76	13.96	14.36	14.69	3.91	1.05
1893 × 2048	3.81	14.02	14.84	14.94	3.92	1.07
3877 × 4096	3.93	15.14	15.15	15.16	3.86	1.00
7682 × 8182	4.32	15.89	16.02	16.11	3.73	1.01
16,364 × 8182	4.54	16.70	16.71	16.71	3.68	1.00

**Figure 8:**
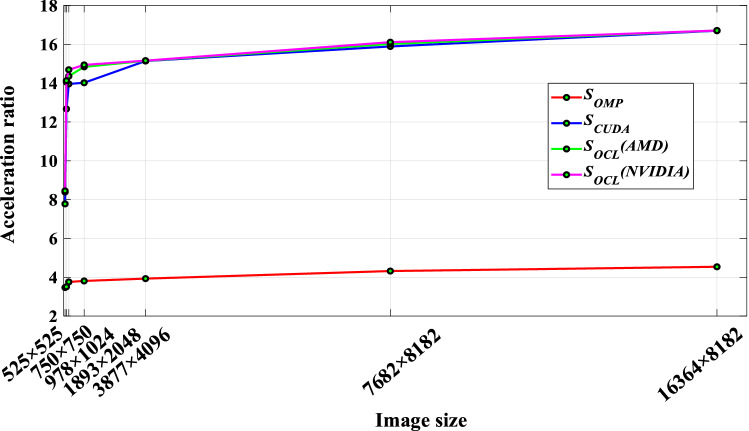
Speedup of parallel algorithms on four platforms.

In order to more intuitively observe the performance improvement of the UM image enhancement parallel algorithm, Fig. 8 is drawn. According to the analysis of the figure, when the image size is between 525 × 525 and 750 × 750, the values of $S_{OMP}$, $S_{CUDA}$, and $S_{OCL}$ all show an increasing trend with the increase of image size, and the slope of the curve is larger. The three parallel algorithms of OMP_UM, CUDA_UM, and OCL_UM all show good data scalability. Obviously, CUDA_UM and OCL_UM have obtained a higher acceleration ratio than OMP_UM. On the one hand, the main memory bandwidth is only one-tenth of the video memory bandwidth. On the other hand, the design of a multi-threaded parallel algorithm takes time in thread startup, synchronization, and scheduling, as well as the limitation of the number of CPU physical cores, $S_{OMP}$ is proportional to the number of CPU cores, and the maximum speedup is 4.54 times. When the image is small, the speedup of CUDA_UM and OCL_UM parallel algorithms increases greatly. When the image size reaches 750 × 750, the parallel scalability of the GPU can be fully expanded. This is mainly because the transmission of data between the host side and the device side is not time-consuming at this time, and the abundant computing resources of the GPU can create sufficient work-items to meet the parallel processing of a large amount of data. With the further increase of image frames, although $S_{CUDA}$ and $S_{OCL}$ still maintain a high value, the OCL_UM parallel algorithm achieves a maximum speedup of 16.71 times when dealing with large images. However, when the image size is more than 750 × 750, the speedup curve shows a gradually flatting trend. It is mainly due to the gradual increase of the time cost of data transmission between the host and the device, which seriously affects the execution efficiency of the algorithm.

**Figure 9:**
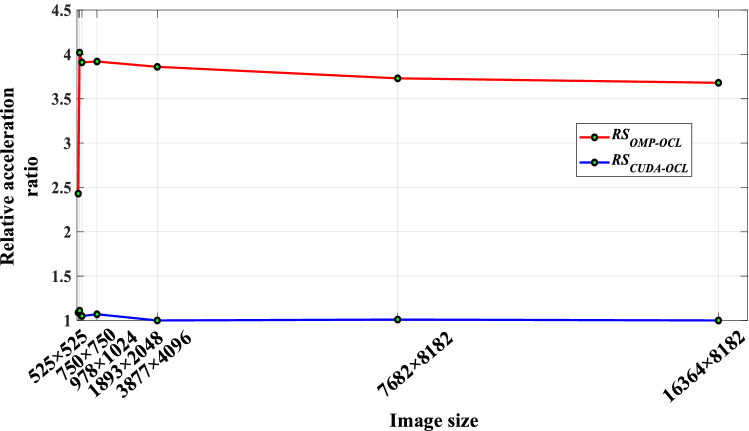
Relative acceleration ratio trend graph.

The acceleration of the UM image enhancement algorithm is different in different parallel architectures. With the increase of the image frame, the speedup of the OMP_UM parallel algorithm increases steadily, but the speedup is only close to the number of CPU cores. On the other hand, the OCL_UM parallel algorithm shows a better acceleration effect on the whole test image set. It can be seen from Fig. 9 that the OCL_UM parallel algorithm can achieve a speedup of 2–4 times compared with the OMP_UM parallel algorithm. Analyzing the reason, the main reason is that the GPU of heterogeneous many-core architecture uses abundant computing resources to start enough work-items to participate in parallel computing when the image size is large.

Figure 9 shows that the $RS_{CUDA - OCL}$ value is between 1.00 and 1.11, indicating that the CUDA_UM and OCL_UM parallel algorithms based on the GPU architecture have achieved an approximate level of acceleration. Although AMD and NVIDIA GPU computing platforms have different architectures, the organization of compute units is also different. However, both of them adopt the hierarchical architecture, and the main optimization techniques and methods used in this paper are also roughly the same. On the other hand, the OCL_UM parallel algorithm can use pre-compiled offline to generate binary files to create program objects and generate executable systems, which saves system execution time.

### Portability analysis of OCL_UM parallel algorithm

In order to verify that the OCL_UM parallel algorithm in this paper has good portability, that is, the scalability of the GPU platform, it can also achieve high performance on GPUs with different computing capabilities. In the platform test, AMD Radeon RX 5700 XT graphics card and NVIDIA Geforce GTX1060 graphics card were selected for OCL_UM parallel algorithm experiments, the running time of the algorithm was counted, and the speedup was calculated, as shown in Tables 4 and 5 respectively. In order to observe the experimental results intuitively, Figs. 7 and 8 are drawn respectively. Through the previous description, it can be seen that the running time and speedup of the OCL_UM parallel algorithm on the two GPU parallel computing platforms are basically the same, indicating that the OCL_UM parallel algorithm has good portability and scalability on the GPU.

#### System performance bottleneck analysis

Through the analysis, it can be known that the UM processing step of the OCL_UM parallel algorithm requires a lot of memory read and write operations. The image data and Gaussian convolution template data need to be read $H^{{2}} n^{{2}}$ times in memory, and the resulting image should be written in memory for $H^{{2}}$ times. Suppose the original image size is 4096 × 4096, and the storage space allocated for each pixel value is 4B, so the total amount of memory access data is about 0.128 GB. Divided by the actual kernel execution time of 0.000943 s, the resulting bandwidth value is about 135.74 GB/s, which is close to the 192 GB/s bandwidth of the GeForce GTX1060 display memory. Therefore, it is obvious that the efficiency of the OCL_UM parallel algorithm is limited by the global memory bandwidth.

As can be seen from Fig. 8, when the image size is small, the operation speed of the OCL_UM parallel algorithm increases faster. However, when the image size is large, the speedup of the OCL_UM parallel algorithm shows a slow downward trend. The main reason is that in the operation of the OpenCL parallel algorithm, the CPU is responsible for reading and outputting image data, but the process is not accelerated. As the size of the processed image increases, the time it takes to read and output the image data also increases. Therefore, the performance bottleneck of the OCL_UM parallel algorithm is the bandwidth of video memory and the bandwidth of data transmission between main memory and video memory.

## Conclusion

In this paper, the parallel computing architecture of OpenCL and the parallelism of the CPU_UM algorithm are analyzed and discussed, and a GPU parallelization implementation method of the pixel-level image processing CPU_UM algorithm is designed. In this paper, an OCL_UM parallel algorithm that supports both coarse-grained and fine-grained parallelism is proposed. The element-level vectorized data parallelism is further discussed, and a new data layout pattern is proposed, which improves the density of the work-item computing in local memory. The experimental results show that the OMP_UM algorithm and the CUDA_UM algorithm, especially the OCL_UM parallel algorithm, can greatly improve the processing speed of image enhancement. The OCL_UM parallel algorithm shows a good acceleration effect on both AMD and NVIDIA parallel computing platforms. With the increase of image size, the speedup of the OCL_UM algorithm shows an upward trend, showing good data scalability, and platform portability. The maximum speedup of the OCL_UM parallel algorithm is 16.71 times, which proves the strong advantage of GPU in digital image parallelization processing. In this paper, single GPU and CPU work together, and the future work is to transplant the OCL_UM algorithm on single GPU to multi-GPU, in order to further optimize the OCL_UM algorithm and tap greater performance improvement potential.

## Data Availability

All data generated or analyzed during this study are included with this published article.
